# Distal protection of endovascular recanalization for symptomatic non-acute occlusion of vertebrobasilar artery

**DOI:** 10.1007/s00701-025-06525-4

**Published:** 2025-04-15

**Authors:** Qiuli Li, Xiaoxi Yao, Yuanbiao Lei, Haipeng Li, Liu Tu, Yi Zhang

**Affiliations:** Department of Neurology, The First People’s Hospital of Chenzhou affiliated to the University of South China, No.102 Guoqing Road, Chenzhou, Hunan 423000 People’s Republic of China

**Keywords:** Distal protection, Endovascular recanalization, Safety and efficacy, Vertebrobasilar artery

## Abstract

**Purpose:**

The research aimed to investigate the safety and efficacy of distal protection of endovascular recanalization for symptomatic non-acute occlusion of the intracranial vertebrobasilar artery.

**Methods:**

8 consecutive patients with symptomatic non-acute VBA from April 2023 to April 2024 who underwent endovascular recanalization were retrospectively analyzed.

**Results:**

8 patients (median age 56 years; mean pretreatment National Institutes of Health Stroke Scale (NIHSS) score 6; 87.5% male) presenting with recurrent transient ischemic attacks(TIAs) (n = 1) or strokes (n = 23) were treated from April 2023 to April 2024. Median time from symptoms onset to treatment was 21 days(range: 10–43). Median time from occlusion confirmed to treatment was 13 days(range:8–26). Among the 8 patients, 8 (100%) achieved successful recanalization. The rate of periprocedural complications was 25%(2/8). Periprocedural complication included one asymptomatic intracranial hemorrhage(asICH) and thrombus translocation. The median follow-up time was 9 months (range: 6–12), with no stroke or TIA. At 90 days, there were one death (unrelated to the procedure) and 75% patients with an available modified Rankin Scale (mRS) score achieved a good outcome (mRS score of 0–2).

**Conclusion:**

The distal protection of stent retriever for endovascular recanalization for symptomatic non-acute occlusion of VBA is technically safe and may decrease procedure-related complications.

## Introduction

The intracranial vertebral artery (VA) and the basilar artery (BA) are the common sites of atherosclerotic occlusion [3, 4, 8, 13], and a subset of patients may still suffer from repeated TIAs and strokes in the non-acute or chronic stage even under aggressive medication [7, 15]. Although posterior circulation accounts for 20% of stroke cases, neurointerventional procedures for secondary prevention in this region have received significantly less research attention compared to anterior circulation interventions [11]. With the improvement of the concept of ischemic prevention and treatment and the progress of endovascular instruments, endovascular recanalization and stenting of non-acute occlusion of intracranial arteries has been increasingly performed. The possible periprocedural complications of endovascular recanalization for non-acute occlusion of the intracranial VBA mainly include ischemic complications such as perforator stroke, vascular dissection, acute thrombosis, distal embolism, and hemorrhagic complications [16, 18–20]. Therefore, endovascular recanalization for patients with non-acute intracranial VBA occlusion remains clinically challenging [5, 14]. There some advantages of the Balloon Angioplasty with the distal protection of Stent Retriever technique, referred to as the distal protection of stent retriever technique was described in acute intracranial artery atherosclerosis-related occlusion, which may be associated with decreasing procedure-related complications and time savings [17]. We aimed to assess the safety and efficacy of the distal protection of endovascular recanalization for symptomatic non-acute occlusion of the intracranial VBA.

## Methods

### Study design and ethics

This retrospective study was approved by the Ethics Committee of Chenzhou No 1 People's Hospital(2,024,017) and performed in accordance with the Declaration of Helsinki.

### Study population

“Non-acute occlusion” was defined as symptomatic (transient ischemic attack [TIA] or stroke), complete occlusion of the intracranial vertebrobasilar artery (VBA), presumed to be of atherosclerotic etiology, where endovascular therapy was performed more than 48 hours after the time when the patients were last seen well [1]. Data from 8 consecutive patients who presented with aggressive ischemic events in the non-acute stage of VBA occlusion and were treated with the distal protection with stent retriever distal protection at our institution between April 2023 to April 2024 were retrospectively reviewed. The study was approved by the Institutional Review Board of the hospital’s ethics committee. Informed consent was obtained from all patients. Eighty-seven point five percent (87.5%) of the subjects were male, with a mean age of 56 years (range 47–67). The inclusion criteria were as follows: (1) intracranial atherosclerosis was the primary etiology; (2) experienced recurrent TIAs or stroke related to occluded VBA despite aggressive medical treatment, which was defined as the treatment including antiplatelet therapy, statins, blood pressure and glucose control, smoking cessation; (3) patients were normally recanalized with dominant VA occlusion together with contralateral occlusion, hypoplasia, or severe stenosis (> 70%) or BA occlusion; and (4) hemodynamic failure were confirmed based on the clinical and imaging evidence. The exclusion criteria were as follows: (1) non-atherosclerotic occlusion, such as vasculitis, arterial dissection, or embolic disease; (2) clinical symptoms were stable with aggressive medical treatment; (3) contraindications to operation, such as known allergy or contraindication to aspirin, clopidogrel, or anesthesia; (4) the distal protection of stent retriever technique was adopted. (5) life expectancy < 1 year because of other medical conditions.

### Endovascular procedures

**Fig. 1:**
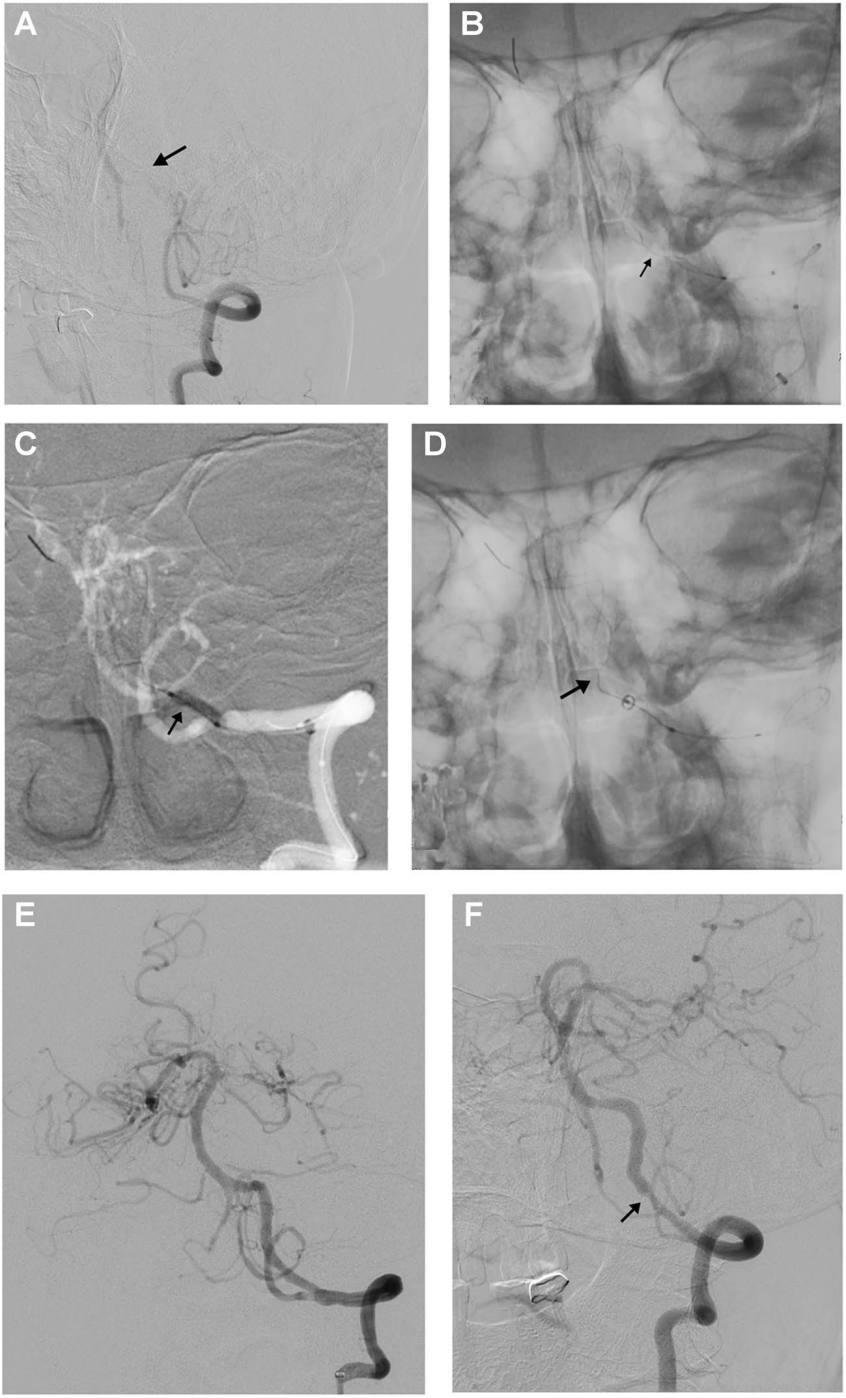
Illustrative case of a patient. Male patient, 56 years old; prestented with dizziness and slurred speech with VA occlusion treated with distal protection for endovascular recanalization.; The admission NIHSS score was 4, and the mRS score was 2. **A**. Digital subtraction angiography showed that the distal segments vertebral artery was occluded. **B**. Digital subtraction angiography displayed Syphonet stent and the occluded segment of vertebral artery. **C**. a suitable-diameter balloon (2.5 mm × 12 mm) was performed to dilate the suspected primary and subsequent thrombosis lesions from the distal to proximal with the distal protection of stent retriever. **D**. After intermediate catheter passed through lesion site, the stent retriever was withdrawn under continuous negative pressure. **E**. Postprocedure angiography revealed that the residual stenosis rate was 20%. **F**. At 9 months the in-stent restenosis was 50%

Combination therapy with oral aspirin (100 mg) and clopidogrel (75 mg) was initiated at least 3 days before endovascular procedures. The procedures were performed under general anesthesia. The right femoral artery was inserted by an 8 F sheath, using the Seldinger technique. Unfractionated heparin at 50 IU/kg was injected intravenously for systemic heparinization. With coaxial technique, the thrombectomy catheter was placed in the area of total occlusion, a 0.014 -inch microguide wire was carefully passed through the occlusion under the guidance of a microguidewire. Angiography was used to confirm that the distal vessel was occluded (Fig. 1A). A Syphonet (Achieva, China) stent was selected as the distal protection device, and the proximal 1/3 of the stent was located at the lesion. After the distal end of the thrombectomy stent was anchored, the microcatheter could be withdrawn directly (Fig. 1B). During the operation, angiography was performed under sufficient pressure to accurately identify the internal components of the occluded segment. If the occluded segment could not be accurately assessed after the microcatheter was withdrawn, a suitable-diameter balloon would be used to dilate the suspected primary and with subsequent thrombosis lesions from the distal to proximal under the guidance of the Syphonet stent with guide wire. Then again angiography was performed through the aspiration catheter to further identify the internal components of the occluded segment. Once detection of intraprocedural distal embolization during the procedure, the aspiration catheter would be advanced to remove the thrombus across the proximal occlusion under continuous negative pressure. The distal protection device of the stent retriever will be withdrawn to remove the thrombus in the stent capture basket. The stent was repositioned at the lesion site, followed by sequential balloon dilation from the distal to proximal segments (Fig. 1C). Finally, the stent was withdrawn after angiography confirmed the absence of thrombus and stable antegrade blood flow (Fig. 1D). After recanalization, antegrade blood flow was assessed according to the modified thrombolysis in cerebral infarction (mTICI) grading, and mTICI ≥ 2b indicated successful recanalization (Fig. 1E). Six months after recanalization, digital subtraction angiography was performed (Fig. 1F), and restenosis was described as ≥ 50% stenosis after stent implantation and ≥ 20% absolute luminal loss at follow-up [10].

Low molecular weight tirofiban 6–8 mL/h (based on the patient’s body weight) was given 12 or 24 h only if intracranial hemorrhage was excluded after operation. Postoperative systolic/diastolic pressures were controlled less than 110/70 mmHg. All patients were advised to take aspirin (100 mg/day) and clopidogrel (75 mg/day) for 6 months with or without subsequent cessation of clopidogrel.

### Data collection

The data of patient included demographics and cardiovascular risk factors, including age, sex, hypertension, diabetes mellitus, hyperlipidemia, previous history of stroke, coronary artery disease and smoking. The median time between symptom onset and occlusion comfirmed were recorded. The mRS scores and NIHSS scores were assessed. Technical procedure complications, clinical follow-up and follow-up angiography were evaluated. Favorable functional outcome was defined as an mRS scores 0–2.

### Statistical analysis

The statistical methods used were descriptive. Quantitative data were expressed as means ± standard deviation or as medians with interquartile range, whereas categorical data were presented as numbers and percentages.

## Results

Eight patients (56 years; range, 47–67 years) with male predominance who were treated with the distal protection of endovascular recanalization were enrolled. The baseline demographic and clinical characteristics are listed in Table 1. Eight patients had acute stroke. Occlusion was located in the intracranial VA in seven (87.5%) patients and BA in one patient (12.5%). All patients had evidence of previous infarction in the VBA distribution on MR imaging. Hypertension was present in seven patients, six patients had diabetes mellitus, one patient had cardiovascular disease, and three patients had smoking history and hyperlipidemia. The duration from symptom onset to treatment was 10–43 days (median 21) and 8–26 days (median 13) from occlusion confirmed to treatment. The overall technical success rate was 100%. Postoperative complications took place in two (25%) patients. Two patients experienced thrombus translocation, which were removed successfully after thrombectomy. One patient recovered well without neurologic impairment and one experienced asymptomatic ICH. Although the patient didn’t suffer a asymptomatic deterioration after asymptomatic ICH, he died of pneumonia and respiratory failure 2 months after discharge. The patients were followed up for a mean of 9 months (range: 6–12). Excluding the patient who died (case 6), the rate of mRS (range: 0–2) scores were 75% at the 90-day follow-up, which was 62.5% higher than before endovascular treatment. Angiographic follow-ups were available for seven patients, in-stent stenosis was 25.7% (10–60%).

**Table 1:** Patients' baseline demographic and clinical characteristics

Sex,male	7(87.5%)
Age,years,median	56(47-67)
Medical History, *n*(%)	
Hypertension	7(87.5%)
Diabetes mellitus	6(75%)
Cardiovascular disease	1(12.5%)
Smoking	3(37.5%)
Hyperlipidemia	3(37.5%)
mRS score on admission, *n*(%)	
0-2	5(62.5%)
3	3(37.5%)
NIHSS,on admission, median(range))	6(2-12)
Symptom onset to treatment,days,median(range)	21(10-43)
Occlusion confirmed to treatment,days,median(range)	13(8-26)
recanalization artery, *n*(%)	
VA intracranial	7(87.5%)
BA	1(12.5%)
technical success, *n*(%)	8(100%)
RSR,median(range)	21%(10-40%)
Complication rate, *n* (%)	2（25%）
Dissection	0（0%）
Perforation	0（0%）
asICH	1（12.5%）
Hyperperfusion syndrome	0（0%）
Thrombus translocation	2（25%）
Follow-up time,months,median(range)	9(6-12)
30-DAY stroke or death, n (%)	1（12.5%）
90-day mRS score,median(range)	
0-2	6(75%)
3	1(12.5%)
ISR on follow-up image,median(range)	25.7%(10-60%)

*NIHSS *National Institutes of Health Stroke Scale, *mRS *modified Rankin Scale, *asICH *asymptomatic intracranial hemorrhage, *BA *basilar artery, *VA *vertebral artery, *ISR *in-stent restenosis rate

## Discussion

Previous studies showed the recanalization of non-acute occlusion of the VBA was a high-risk procedure [6]. The recanalization and stenting for non-acute occlusion of the VBA has generally been used in clinical practice but the rates of perioperative complications were also high [9], such as cerebral hemorrhage, dissection, acute reocclusion and thrombus disruption and translocation. During the recanalization, the major technical challenge is traversing the occlusion site with a guidewire. Since there were fewer side and perforating branches in the intracranial VA, it was easier to result in blood flow retardation proximal to the occlusion and subsequent thrombus formation, leading to longer segments of occlusion in the VA [21], which make it more difficult to cross. It was reported that the application of the distal protection in treating acute intracranial artery atherosclerosis-related occlusion may increase the rates of successful reperfusion and decrease procedure-related complications [17]. In this study, all patients were treated with the distal protection of endovascular recanalization. It is more likely to go through the occlusion site after balloon angioplasty at the stenotic site, which may explain high technique success in our case series. Meanwhile, a thrombus may be dislodged distally when crossing the occluded segment during recanalization. Embolization is a well-known and serious complication of endovascular therapies. In order to avoid embolism, distal protection devices have been developed and are currently only being used widely in carotid artery stenting (CAS) procedures [2]. In fact, the management of high-burden thrombus in intracranial VA is more tricky. Once the clot migration in VA occlusion occured during the procedures, the consequence was lethal. Currently, there was no report about the distal protection for endovascular recanalization of symptomatic non-acute occlusion of VBA. As the application of distal protection, the rates of perioperative complications decreased. Though two patients experienced thrombus translocation in our study, but the thrombus were removed successfully. This is the first case series to evaluate the significance of the distal protection for endovascular recanalization involving VBA occlusion. Reducing the frequency of instrument exchanges, the risk of complications can be minimized. By using the retrieval basket of the Syphonet thrombectomy stent as protection, the distal protection of stent retriever technique can reduce the risk of thrombus disruption and translocation caused by balloon dilatation.

## Limitations

 Besides the sample size is too small, there were other limits in our findings. Considering that the occlusion time of VBA in these patients was relatively short and the thrombus had not yet fully organized, it was hypothesized that the microguidewire might pass through more easily [12]. Therefore, we did not perform high-resolution MRI for this group of patients.

## Conclusion

Our case series indicates that the use of distal protection with a stent retriever during endovascular recanalization for symptomatic non-acute occlusion of the vertebral basilar artery (VBA) is technically feasible and may potentially reduce procedure-related complications.

## Data Availability

No datasets were generated or analysed during the current study.
